# The hidden costs of caring: mapping pathways to depression among informal caregivers in Europe

**DOI:** 10.1093/geroni/igaf122.2167

**Published:** 2025-12-31

**Authors:** Marco Albertini, Eva Bei

**Affiliations:** University of Bologna, Bologna, Emilia-Romagna, Italy; University of Bologna, Bologna, Emilia-Romagna, Italy

## Abstract

Europe is experiencing a demographic shift characterized by increasing longevity and declining birth rates, leading to rising long-term care (LTC) demands. As informal caregivers become the backbone of LTC systems, understanding the societal pathways linking caregiving intensity to mental health outcomes such as depression becomes crucial. Using data from the European Social Survey (ESS) rounds 7 and 11, this study employed Structural Equation Modeling (SEM) with maximum likelihood estimation to examine pathways between socioeconomic status (modeled as a latent construct with education, income, and employment indicators), caregiving intensity, and depression. We analyzed 23,799 individuals reported providing informal care. The caregiving stress process model framed our study, with care intensity hypothesized as a key mediator between SES and depression. SEM revealed significant associations between key SES indicators and caregiving patterns. Lower household income emerged as the strongest SES indicator of care intensity (β=-0.690, p<.001), followed by unpaid work status (β=-0.502, p<.001) and lower levels of educational attainment (β=-0.476, p<.001). Gender differences were pronounced, with women reporting higher levels of both caregiving intensity (β = 0.069, p<.001) and depression (β = 0.085, p<.001). SEM analysis confirmed caregiving intensity as a significant predictor of depression (β = 0.070, p<.001), supporting its mediating role between all SES predictors and depression. Additionally, satisfaction with healthcare services negatively predicted both caregiving intensity (β=-0.086, p<.001) and depression (β=-0.106, p<.001), highlighting healthcare system importance for caregivers. These findings contribute to understanding how socioeconomic determinants stratify care intensity as a mediator pathway to depression, offering valuable insights for policymakers to support informal care across Europe.

